# Anatomic changes after repair of traumatic bilateral diaphragmatic rupture impede bi-caval dual lumen catheter insertion for veno-venous extracorporeal membrane oxygenation treatment

**DOI:** 10.1186/s13054-017-1667-4

**Published:** 2017-04-04

**Authors:** Alexa Hollinger, Daniel Tuchscherer, Jens Bremerich, Martin Siegemund

**Affiliations:** 1 Department for Anaesthesia, Surgical Intensive Care, Prehospital Emergency Medicine and Pain Therapy, University Hospital Basel, Spitalstrasse 21, CH-4031 Basel, Switzerland; 2 Department of Radiology and Nuclear Medicine, University Hospital Basel, Spitalstrasse 21, CH-4031 Basel, Switzerland

Extracorporeal membrane oxygenation (ECMO) is a therapeutic option used increasingly in the treatment of severe acute respiratory distress syndrome (ARDS). Choosing an adequate cannula type and insertion site can be a challenge. The insertion of a bi-caval dual lumen (Avalon®) catheter in the superior vena cava instead of two venous single-lumen catheters facilitates mobilisation and physiotherapy of patients, and hence is being used more and more [1].

A middle-aged patient was admitted to our hospital after severe multiple trauma. Before admission to our hospital, damage control surgery including bilateral diaphragmatic repair and ileotransversostomy was performed.

The postoperative course was complicated by disseminated intravascular coagulation (DIC). Six days after the accident, the patient could be stabilized to be eligible for transportation to the hospital by an air rescue service. The patient was transferred directly to the Surgical ICU under controlled mechanical ventilation.

Within the first 24 h after admission, the respiratory function deteriorated to ARDS. Advanced respiratory support, including veno-venous ECMO, was applied to sustain gas exchange in the hope it could improve survival. Because of the underlying complex abdominal trauma we tried to insert a bi-caval dual lumen catheter into the right jugular vein. Due to surgical reconstruction of the bilateral diaphragmatic rupture and consecutive anatomical changes, several attempts to place either the guide wire or the catheter tip into the inferior vena cava (IVC) under transthoracic and transoesophageal echocardiography visual guidance failed; both guide wire and dual lumen catheter could not bypass the right ventricle to the IVC.

Therefore, we decided to insert two single lumen catheters into the right jugular and femoral vein, whereupon ECMO treatment could be performed without further technical problems. In a post-hoc reconstruction of the thoracic computed tomography (CT) scan we discovered an altered path of IVC transition into the right atrium following surgical repair of the bilateral diaphragmatic rupture (Fig. 1)*.*

**Fig. 1:**
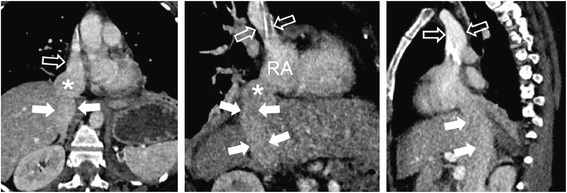
Paracoronal reconstructions of contrast-enhanced CT show normal connection of the superior vena cava (*open arrows*) into the right atrium (*RA*). Connection of the IVC (*arrows*) to the RA, however, is abnormal with angulation and bulbus-shaped configuration (*)

In patients with right-sided diaphragmatic rupture and surgical reconstruction we recommend a three-dimensional reconstruction based on three-dimensional echocardiography or CT of the venous inflow to the right atrium before attempting to insert a bi-caval dual lumen catheter [2]. Notwithstanding that the manufacturer recommends insertion of the guide wire under angiographic control, we assume that use of fluoroscopy most likely would have been associated with the same difficulties.

## Abbreviations

ARDS: Acute respiratory distress syndrome
CT: Computed tomography
DIC: Disseminated intravascular coagulation
ECMO: Extracorporeal membrane oxygenation
IVC: Inferior vena cava
